# Behavior Change After 20 Months of a Radio Campaign Addressing Key Lifesaving Family Behaviors for Child Survival: Midline Results From a Cluster Randomized Trial in Rural Burkina Faso

**DOI:** 10.9745/GHSP-D-15-00153

**Published:** 2015-11-03

**Authors:** Sophie Sarrassat, Nicolas Meda, Moctar Ouedraogo, Henri Some, Robert Bambara, Roy Head, Joanna Murray, Pieter Remes, Simon Cousens

**Affiliations:** ^a^​ Centre for Maternal Adolescent Reproductive and Child Health (MARCH), London School of Hygiene and Tropical Medicine, London, UK; ^b^​ Centre Muraz, Bobo Dioulasso, Burkina Faso; ^c^​ Africsanté, Bobo Dioulasso, Burkina Faso; ^d^​ Direction Générale des Études et des Statistiques Sectorielles (DGESS), Ministère de la Santé, Ouagadougou, Burkina Faso; ^e^​ Development Media International, London, UK; ^f^​ Development Media International, Ouagadougou, Burkina Faso

## Abstract

**Background:**

In Burkina Faso, a comprehensive 35-month radio campaign addressed key, multiple family behaviors for improving under-5 child survival and was evaluated using a repeated cross-sectional, cluster randomized design. The primary outcome of the trial was postneonatal under-5 child mortality. This paper reports on behavior change achieved at midline.

**Method:**

Fourteen community radio stations in 14 geographic areas were selected based on their high listenership. Seven areas were randomly allocated to receive the intervention while the other 7 areas served as controls. The campaign was launched in March 2012. Cross-sectional surveys of about 5,000 mothers of under-5 children, living in villages close to the radio stations, were conducted at baseline (from December 2011 to February 2012) and at midline (in November 2013), after 20 months of campaigning. Statistical analyses were based on cluster-level summaries using a difference-in-difference (DiD) approach and adjusted for imbalances between arms at baseline. In addition, routine health facility data were analyzed for evidence of changes in health facility utilization.

**Results:**

At midline, 75% of women in the intervention arm reported recognizing radio spots from the campaign. There was some evidence of the campaign having positive effects on care seeking for diarrhea (adjusted DiD, 17.5 percentage points; 95% confidence interval [CI], 2.5 to 32.5; *P* = .03), antibiotic treatment for fast/difficult breathing (adjusted DiD, 29.6 percentage points; 95% CI, 3.5 to 55.7; *P* = .03), and saving money during pregnancy (adjusted DiD, 12.8 percentage points; 95% CI, 1.4 to 24.2; *P* = .03). For other target behaviors, there was little or no evidence of an impact of the campaign after adjustment for baseline imbalances and confounding factors. There was weak evidence of a positive correlation between the intensity of broadcasting of messages and reported changes in target behaviors. Routine health facility data were consistent with a greater increase in the intervention arm than in the control arm in all-cause under-5 consultations (33% versus 17%, respectively), but the difference was not statistically significant (*P* = .40).

**Conclusion:**

The radio campaign reached a high proportion of the primary target population, but the evidence for an impact on key child survival-related behaviors at midline was mixed.

## Abstract (precis)

The radio campaign reached a high proportion of mothers, but the impact on self-reported behaviors at midline was mixed. Some reported episodic behaviors such as care seeking for diarrhea and obtaining treatment for fast/difficult breathing improved more in intervention than control areas, but there was little or no difference between areas in reported habitual behaviors, such as exclusive breastfeeding, complementary feeding, hand washing with soap, and use of bed nets.

## BACKGROUND

The number of under-5 deaths worldwide has been reduced by 50%, from 12.7 million in 1990 to 6.3 million in 2013.^1^ Still, under-5 mortality risk remained above the Millennium Development Goal (MDG) target in 52 of the 75 countries that account for more than 95% of child deaths.^1^

A number of interventions are known to be effective in preventing under-5 child deaths.^2^ At present, however, many effective interventions do not reach the children who need them, and none of the 75 countries mentioned above has yet achieved anything close to full population coverage for even a minimum set of essential interventions.^3^ Poor coverage has been attributed to weaknesses in both provision of and demand for services.^4^^,^^5^

While much effort toward achieving the MDGs has been focused on the health system and the supply side,^6^ less attention has been placed on increasing demand for services. It is now acknowledged, however, that behavior change plays an important role in enhancing child survival in low- and middle-income countries.^7^

Behavior change interventions encompass a wide range of approaches including interpersonal-based, community-based, media, and social marketing approaches.^8^ Mass media campaigns have the potential to reach a large audience at relatively low cost compared with other behavior change approaches. A review of evaluations of mass media health campaigns,^9^ most of which addressed tobacco control and lifestyle behaviors in high-income countries, concluded that targeted, well-executed mass media campaigns can have small to moderate effects not only on knowledge, beliefs, and attitudes but also on behaviors. A more recent review, focused on mass media interventions for child survival-related behaviors in low- and middle-income countries, concluded that “media-centric” campaigns can positively impact a wide range of child health behaviors, although the authors acknowledged likely publication bias toward successful campaigns.^10^

In Burkina Faso, Development Media International (DMI) implemented a 35-month community radio campaign, using the “*Saturation*+ methodology,” to address key family behaviors for improving under-5 child survival. An overview of the *Saturation+* methodology is given elsewhere,^11^ and further details about the methodology and lessons learned during implementation of the DMI radio campaign are provided in a companion article in *Global Health: Science and Practice*.^12^ The campaign was evaluated using a repeated cross-sectional, cluster randomized design. Community radio stations were chosen as the delivery channel for the campaign as they are widely listened to in those rural areas where child mortality is highest and because, with their limited transmission range, a randomized design was possible. The use of television, which is broadcast nationally, would have made a randomized design difficult, if not impossible.

The primary objective of the trial was to investigate whether the *Saturation+* approach to designing and implementing a mass media campaign can change behaviors on a scale large enough to result in measurable reductions in all-cause, postneonatal under-5 child mortality. To this end, household surveys were conducted in all clusters at 3 time points: at baseline, at midline, and at endline. At midline, the objective of the trial was to measure coverage of the campaign and to investigate changes in self-reported behavior achieved after 20 months of campaigning. The purpose of this article is to report on the midline results. Mortality reduction as well as behavior change achieved at endline will be reported separately when results are available.

An innovative cluster randomized trial investigated whether a mass media campaign can change child survival-related behaviors at scale.

## METHODS

### Setting

The population of Burkina Faso was estimated at 15.7 million people in 2010, of whom 77% lived in rural areas.^13^ Since 1990, the under-5 mortality rate has declined from an estimated 202 deaths per 1,000 live births to 186 deaths per 1,000 live births in 2000 and to 98 deaths per 1,000 live births in 2013.^1^ In 2013, malaria, pneumonia, and diarrhea accounted for an estimated 23%, 15%, and 10% of under-5 child deaths, respectively.^14^

The government is the main health service provider, and the country is organized into 70 health districts, each with 1 district hospital and 10 or more primary health facilities (Centre de Santé et de Promotion Sociale, or CSPS). The Integrated Management of Childhood Illness (IMCI) strategy was introduced in 2003.^15^ Since 2002, free antenatal care (ANC) has been offered in public health facilities, and in 2006 subsidies were introduced for child birth and emergency obstetric care.^16^ In 2005, artemisinin-based combination therapy (ACT) replaced chloroquine as the recommended treatment for uncomplicated malaria, and in 2010 ACT was introduced at the community level.^17^

### Cluster Identification, Definition, and Randomization

In early 2011, we identified 19 distinct geographical areas using digital terrain maps and an engineer’s modeling together with on-the-ground mapping of radio signal strength. Each geographical area contained one or more community FM radio stations, with little or no overlap of radio signal between areas.

We then performed a cross-sectional survey in each geographic area to assess women’s radio listenership. Fourteen areas with high levels of reported listenership (above 60% of women listening to the radio in the past week) were selected for inclusion in the trial, and, within each area, the radio station with the highest listenership was chosen as a potential partner to implement the campaign. High radio listenership was a key factor for the power of the trial given our assumption that the effect of the campaign would be directly proportional to the number of women listening to the radio.

**FIGURE 1.**
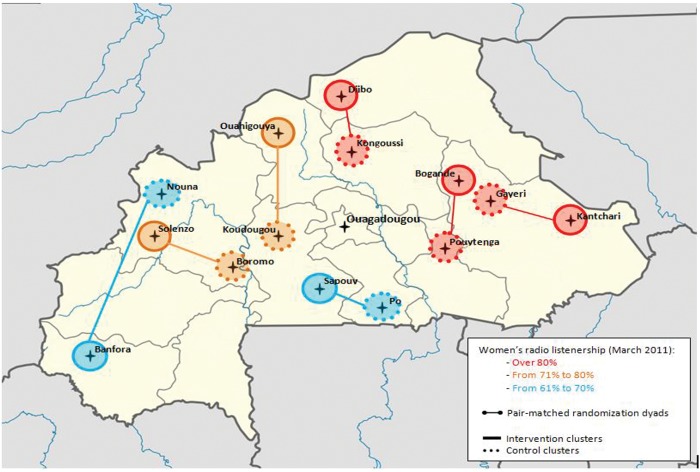
Pair-Matched Randomization of Clusters Based on Geography and Radio Penetration Rate Adapted from Wikipedia.

Seven areas were then randomly allocated to receive the intervention and 7 other areas to serve as controls using pair-matched randomization based on geography and radio penetration rate (Figure 1). Specifically, we defined 3 radio listenership strata (from 61% to 70%, from 71% to 80%, and above 80%), and within each stratum, we paired the areas geographically closest to each other, one of which was randomly assigned to receive the intervention. Randomization was performed by SS and SC (both with the London School of Hygiene and Tropical Medicine), independently of DMI. Due to time constraints with implementing the campaign, randomization was performed before the baseline survey (see below) and therefore could not make use of behavioral and mortality data from the baseline survey. After randomization, DMI began formative qualitative research and capacity building with radio stations in the intervention clusters while the baseline survey took place. Broadcasting started at the end of the baseline survey.

For the purpose of the evaluation, the trial population in each area was restricted to the communities with limited access to television, who would consequently be more likely to listen to the radio. We therefore excluded the population living in the electricity grid, i.e., those living in the towns where the selected control and intervention community radio stations were located, as well as those living in villages within 5 km of the town, in villages with electricity, or in villages with a population above 5,000 inhabitants (and likely to be a priority for the national electrification program). Villages with poor radio signal strength were also excluded.

Using the last national census, we then identified sufficient eligible villages to provide a total population of about 40,000 inhabitants per trial cluster. The average number of villages per cluster was 34 and 29 in the control and intervention arms, respectively. With the exception of Kantchari intervention cluster (toward the East), the town with the community radio station was also the location of the regional or district hospital. The trial population also had access to primary health facilities in villages across each area. The trial was designed to detect, with a statistical power of 80%, a 20% reduction in all-cause, postneonatal under-5 child mortality.

### Brief Description of the Intervention

DMI’s radio campaign launched in March 2012 and ended in January 2015. Women of reproductive age and caregivers of children less than 5 years old were the primary target of the campaign, which covered a wide range of behaviors along the continuum of care (Table 1). A full description of the theory of change—the *Saturation*+ methodology —used to design the campaign and its implementation is provided elsewhere.^11^^,^^12^ Briefly, short spots of 1-minute duration were broadcast in the predominant local language approximately 10 times per day, and interactive long-format programs of 2-hours’ duration were broadcast 5 days per week. The spots were designed to be entertaining and informative and were developed and pretested based on qualitative formative research. Behaviors covered by spots changed weekly, while the long-format program changed daily, covering 2 behaviors a day.

The radio campaign in Burkina Faso broadcast both short spots and longer dramas.

**TABLE 1:** Target Behaviors and Broadcasting Intensity Up to the Month Preceding the Midline Survey (October 2013)

Target Behaviors	No. of Weeks for Short Spots	No. of Long-Format Modules
**Maternal health**		
4 or more ANC visits	3	32
Saving money during pregnancy	3	32
Health facility delivery	5	25
**Newborn health**		
Breastfeeding initiation within 1 hour after birth	6	25
First bath delayed for 24 hours or more after birth in low birth weight infants	1	7
**Child nutrition**		
Exclusive breastfeeding in 0- to 5-month-old children	5	51
Complementary feeding in 6- to 11-month-old children	4	29
Growth monitoring in 0- to 23-month-old children	4	20
**Health care seeking for childhood illnesses**		
Health care seeking for fever	10	41
Health care seeking for pneumonia	7	43
Health care seeking for diarrhea	12	79
**Diarrhea home treatment**		
ORS or increase in fluids for diarrhea	12	79
**Bed net use**		
Bed net use among under-5 children and pregnant women	6	85
**Sanitation**		
Household latrine ownership	2	68
Safe disposal of children's stool	3	68
Hand washing with soap	8	55

Abbreviations: ANC, antenatal care; ORS, oral rehydration solution.

At the time of the midline survey, no radio campaigns of comparable intensity were being broadcast in any of the clusters included in the trial. Various nutrition and sanitation programs were operating in similar numbers of clusters per arm, and community case management for malaria, pneumonia, and diarrhea was supported by the United Nations Children’s Fund (UNICEF) in one of the intervention clusters and one of the control clusters (Table 2).

**TABLE 2:** Other Major Programs Implemented in Intervention and Control Clusters at Time of DMI Radio Campaign Midline Survey (November 2013)

Cluster	Programs/Organizations by Health Sector		
	Nutrition	Sanitation	Community Case Management
**Intervention clusters**			
Banfora	El Mundo	–	–
Bogande	NUTRIFASO program; GRET; Action Contre la Faim (ACF); Programme Alimentaire Modial (PAM)	SaniFaso program	–
Djibo	Croix Rouge (Red Cross)	Oxfam	–
Kantchari	ACF; PAM	ACF	–
Ouahigouya	Terre des Hommes	–	UNICEF
Sapouy	–	–	–
Solenzo	–	–	–
**Control clusters**			
Boromo	–	SaniFaso program; WaterAid	–
Gayeri	NUTRIFASO program; PAM	SaniFaso program	–
Kongoussi	The Hunger Project	Plan International	UNICEF
Koudougou	PAM	–	–
Nouna	UNICEF	–	–
Po	–	–	–
Pouytenga	–	Plan International	–

### Behavioral Surveys

Cross-sectional surveys were performed in all clusters at 3 time points: at baseline, from December 2011 to February 2012, before the launch of the campaign; at midline, in November 2013, after 20 months of campaigning; and at endline, between November 2014 and April 2015, at the end of the campaign. (Endline results will be reported separately.)

#### Sampling

At baseline, the behavioral survey was part of a larger survey conducted to estimate under-5 child mortality during the 2 years prior to the intervention. Due to cost constraints, the baseline survey was conducted in a simple random sample of half the villages included in each cluster. The average number of villages sampled per cluster were 17 and 15 in the control and intervention arms, respectively, with average populations per village of 1,359 inhabitants (range: 55 to 4,730) and 1,430 inhabitants (range: 83 to 4,702), respectively. In the sampled villages, a census was performed of all compounds to identify all women aged 15 to 49 years old and to collect pregnancy history data. The behavioral questionnaire was then addressed to a random subsample of about 5,000 mothers with at least one under-5 child living with them.

At midline, about 5,000 mothers were selected using a 2-stage sampling procedure. In each cluster, 9 villages were first drawn with probability proportional to size from villages surveyed at baseline. In each village, 100 women were then selected by simple random sampling using the census data collected at baseline, and the first 40 eligible and available women were interviewed.

The sample size of 5,000 mothers at each survey was calculated assuming a design effect of 2 with a view to providing an absolute precision of ±3% or better for behaviors relating to all children. The expected precision for behaviors related to childhood illness was ±6% for fever or diarrhea and ±10% for fast or difficult breathing.

#### Questionnaires

At baseline, a short interview with the household head addressed socioeconomic status and radio ownership. Interviews with women addressed their basic demographic characteristics, radio listenership, and family behaviors of relevance to child survival. Questions regarding maternal health referred to the last pregnancy of more than 6 months’ duration, and those regarding newborn health referred to the last live birth. Questions regarding nutrition, health care seeking for childhood illnesses, bed net use, and sanitation applied to the youngest child less than 5 years old. Illnesses were recorded using a recall period of 2 weeks preceding the interview.

At midline, socioeconomic status was not reassessed, and interviews with women used the same baseline questionnaire but with additional questions on radio ownership and recognition of the campaign. Spots broadcast in the last 2 weeks of October were played at the end of the interview, and women were asked whether they had listened to the long-format program by referring to its title. In the control clusters, the same method of recall was used with spots, and the title of the long-format program broadcast in the closest intervention cluster with the same language was mentioned. Interviews were performed using Trimble Juno SB Personal Digital Assistants (PDA). Quality of data collection was monitored regularly, and repeat interviews were requested in cases of missing and/or inconsistent responses.

### Routine Health Facility Data

Routine health facility data were obtained to complement self-reported data on service-dependent behaviors. The “Direction Générale des Etudes et des Statistiques Sanitaires” (DGESS) of the Ministry of Health of Burkina Faso provided monthly absolute numbers of pregnant women attending ANC, health facility deliveries, and all-cause under-5 child consultations in primary health facilities located in the trial clusters for 2011 and 2013.

### Analysis

#### Change From Baseline in Self-Reported Behaviors

Analyses were performed on cluster-level summaries using a difference-in-difference (DiD) approach.^18^^-^^20^ With fewer than about 15 clusters per arm, cluster-level analyses are preferable to methods based on individual-level data.^19^ While generalized estimating equations (GEE) and random effects models have good asymptotic properties, they may not be robust when the number of clusters is small. The GEE approach tends to result in inflated type I errors in such situations,^18^^,^^20^ while the distributional assumptions of random effects models are difficult to verify without a large number of clusters.^18^

For each target behavior (Table 1), in each cluster, the reported prevalence was estimated at baseline and midline, and the difference in prevalence between surveys calculated. The campaign began broadcasting in March 2012, so analyses of maternal and newborn-related behaviors at midline were restricted to pregnancies ending after June 2012 (thus allowing for at least 3 months’ exposure to the campaign). Linear regression was used to regress cluster-level differences in prevalence between surveys on the cluster-level baseline prevalence and the intervention status of clusters (intervention/control). The coefficient of the intervention variable thus provided an estimate of the DiD. Two-sided *t* tests were performed to test the null hypothesis of no intervention effect. Adjustment for cluster-level baseline prevalence was used to account for the phenomenon of regression to the mean.^19^ In the absence of accurate estimates of the intraclass correlation coefficient ρ, weighted analyses may be less efficient than unweighted analyses.^19^^,^^21^ All clusters were therefore given equal weight in the analysis, although the effective sample size in each cluster varied for behaviors applying to a subsample of women and their children (e.g., health care seeking and treatment). The matching procedure used for randomization was ignored as recommended for trials with fewer than 10 clusters per arm.^22^

At midline, a third of women in the Gayeri control cluster (North-East) reported listening to the campaign’s radio station partner in the Bogande intervention cluster (Figure 1). All analyses were performed both on an intention-to-treat and per-protocol basis, the latter excluding all women interviewed in villages where contamination occurred.

#### Adjustment for Confounder Score

At baseline, the mean postneonatal under-5 mortality risk during the 2 years preceding the intervention was estimated at 113.1 per 1,000 children in the intervention arm versus 84.1 per 1,000 children in the control arm, a risk difference of 29.0 deaths per 1,000 children between arms.

To control for imbalance between arms, a confounder score was developed and used to obtain adjusted DiD estimates. Three covariates, particularly imbalanced between arms at baseline and expected to predict mortality, were combined using principal components analysis to produce a single cluster-level summary confounder score. These 3 covariates were the mean distance to the capital, as a proxy for general level of development (158 km versus 232 km in the control and intervention arms, respectively); the median distance to the closest health facility (2.5 km versus 6.3 km, respectively); and the baseline health facility delivery prevalence (81.8% versus 56.0%, respectively). After controlling for the confounder score, the mortality risk difference between arms at baseline was reduced from 29.0 to 4.1 per 1,000 children.

#### Analyses Restricted to Regular Listeners

Regular listeners were defined at baseline and at midline as women who reported listening to the radio in the past 7 days. A sensitivity analysis, restricted to these women, was performed using the methods described above.

#### Dose-Response Analyses

Three categories of radio ownership were defined to look for evidence of effect modification: no radio in the compound, radio in the compound, and radio in the household. In each cluster, the change in reported behavior prevalence from baseline was calculated by radio ownership category. A DiD analysis was performed including an interaction term between intervention status and radio ownership category. Cluster-specific random effects were included to account for the expected correlation in the change from baseline estimated for each radio ownership category in the same cluster.

To examine the relationship between broadcasting intensity and reported behavior change, DiDs for all target behaviors were plotted against broadcasting intensity. Intensity was measured as the number of weeks during which spots were broadcast from March 2012 to October 2013 and as the number of long-format modules during the same period. DiDs were then regressed on broadcasting intensity. The assumption that behaviors are independent of each other may not be true, and, therefore, no formal statistical tests were performed. The 95% confidence intervals (CIs) for the regression coefficients should be interpreted with caution as they may be too narrow.

#### Change From Baseline in Routine Health Facility Data

For each target service (ANC, deliveries, and all-cause under-5 child consultations), the absolute number of consultations at primary health facilities located in the trial clusters was calculated by year and by cluster. For each cluster, the ratio of the absolute number of consultations in 2013 over the absolute number in 2011 was then calculated, and a 2-sided *t* test was used to compare the mean ratio by arm.

### Ethics

The study was approved by the ethical committees of the Ministry of Health of Burkina Faso and the London School of Hygiene and Tropical Medicine. The nature of the intervention precluded formal blinding of respondents and interviewers. Each interviewed woman recorded into the PDA her written consent to participate in the survey, which they were told was about their children’s health, without any mention of the radio campaign. The trial is registered at ClinicalTrials.gov (Identifier: NCT01517230).

## RESULTS

At baseline, the census recorded 19,565 compounds, 40,156 households, and 47,737 women aged 15 to 49 years old in the sampled villages. Among women, 4% were absent at the time of the baseline survey, and 0.1% refused to participate. In total, 5,043 mothers were interviewed about their behaviors across the 14 clusters.

At midline, 8,098 women recorded in the baseline census were visited during the survey. In contrast to baseline, time for fieldwork in each village was much shorter and a higher proportion of women (20%) were absent the day of the visit (23% versus 17% in the control and intervention arms, respectively). Only 0.2% of women who were present refused to participate, 2% were less than 15 years old or more than 49 years old, and 18% did not have a child less than 5 years old; therefore, a total of 5,182 mothers were interviewed. The per-protocol analysis excluded 252 women from villages in Gayeri cluster where contamination occurred at midline.

### Baseline Sociodemographic Characteristics and Self-Reported Behavior Prevalence

While several sociodemographic characteristics of interviewed mothers were similar across arms at baseline, there were some important differences (Table 3). In each arm, about 80% of mothers had lived 5 years or more in their village, their average age was 28 years, and nearly all were married, of whom 40% were in a polygamous union. Around 40% had 2 or more children aged less than 5 years old. The mean age of their youngest child was about 20 months. More Muslims and fewer Catholics/Protestants lived in the intervention arm than in the control arm (Muslims: 60% versus 47%, respectively; Catholics/Protestants: 26% versus 45%, respectively). The Mossi were the largest ethnic group in each arm, but other ethnicities varied across clusters. Only 16% and 10% of women in the control and intervention arms, respectively, had attended school. Households in the control arm tended to have higher socioeconomic status compared with the intervention arm. These sociodemographic characteristics remained stable at midline (Table 3).

**TABLE 3:** Baseline Sociodemographic Characteristics, Distance to Health Facility, and Radio Ownership Among Sampled Women

	Control Arm^a^		Intervention Arm^b^	
	Baseline (N = 2,567)	Midline (N = 2,586)	Baseline (N = 2,476)	Midline (N = 2,596)
**Sociodemographic characteristics**				
Age, mean, y	28.9	30.0	28.4	29.2
Resident for 5 years or more in the village, %	80.3	88.2	83.6	86.4
Ethnicity, %				
Mossi	42.1	43.6	30.1	32.2
Gourmantche	11.5	12.3	26.9	27.1
Gourounssi	22.1	23.7	3.2	1.8
Peulh	6.5	5.7	17.0	16.6
Gouin/Karaboro/Turka	0.2	0.1	13.9	13.9
Marka/Dafing/Dioula	8.4	7.0	3.5	3.3
Bwaba/Bobo	7.5	6.0	3.3	3.6
Other	1.6	1.7	2.1	1.6
Religion, %				
Muslim	47.2	43.4	60.1	59.5
Catholic/Protestant	45.0	49.5	26.4	28.0
Animist	7.8	7.1	13.5	12.5
School attendance, %	15.6	15.7	10.2	8.4
Income-generative activity in the past 2 weeks, %	34.9	39.0	29.3	31.8
Household socioeconomic status, %^c^				
1 (poorest)	14.2	–	18.8	–
2	16.6	–	20.6	–
3	19.3	–	20.2	–
4	21.8	–	20.1	–
5 (least poor)	28.1	–	20.3	–
Two or more under-5 children, %	39.4	35.5	46.4	42.8
Married, %	97.1	98.0	98.3	99.0
Polygamous union, %	39.6	39.7	40.3	44.2
**Distance to the closest health facility, %**				
<2 km	39.5	47.1	18.3	16.3
2–5 km	33.2	28.9	28.2	26.1
>5 km	27.4	24.0	53.4	57.6
**Radio ownership, %**				
No radio	20.5	35.1	13.2	21.9
Radio in the compound	16.7	18.4	22.3	26.2
Radio in the household	62.8	46.4	64.5	51.9

a At baseline: 36 missing values for age; 13 for number of children; 10 for ethnicity; 9 for socioeconomic status; 5 for residence, religion, school attendance, income-generative activity, and marital status; 8 for radio ownership. At midline: 4 missing values for radio ownership; 3 for age; 2 for religion and marital status; 1 for number of children, school attendance, and income-generative activity.

b At baseline: 23 missing values for age; 18 for socioeconomic status; 17 for number of children; 13 for radio ownership; 9 for religion; 7 for ethnicity; 6 for residence, school attendance, income-generative activity, and marital status. At midline: 3 missing values for radio ownership; 1 missing value for religion.

c Household socioeconomic status was measured only at baseline.

**FIGURE 2.**
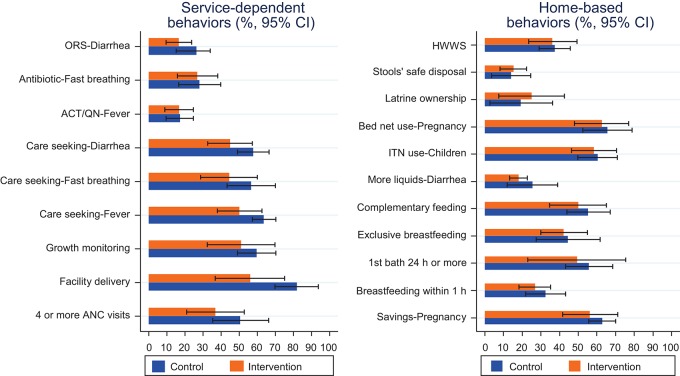
Prevalence of Target Behaviors at Baseline Abbreviations: ACT/QN, artemisinin-based combination therapy/quinine; ANC, antenatal care; CI, confidence interval; HWWS, hand washing with soap; ITN, insecticide-treated bed net; ORS, oral rehydration solution.

At baseline, most service-dependent behaviors tended to be reported more commonly in the control arm than in the intervention arm (Figure 2), perhaps reflecting the difference in access to facilities between the 2 arms, with 40% of women in the control arm living less than 2 km away from a health facility compared with only 18% in the intervention arm (Table 3). In each arm, the proportion of sick children reported to have received treatment was quite low: a third or fewer children suffering from fever, fast/difficult breathing, or diarrhea received the appropriate treatment. Reported home-based behaviors at baseline were more similar between arms, though still tending to be better in the control arm (Figure 2). Early breastfeeding initiation and sanitation-related behaviors such as latrine ownership and safe disposal of stools were reported to be low at baseline, around a third or less. Other home-based behaviors, including saving money during pregnancy, exclusive breastfeeding, complementary feeding, and bed net use, were more common, reported by around 40% to 60% of mothers.

### Reach of the Radio Campaign

**FIGURE 3.**
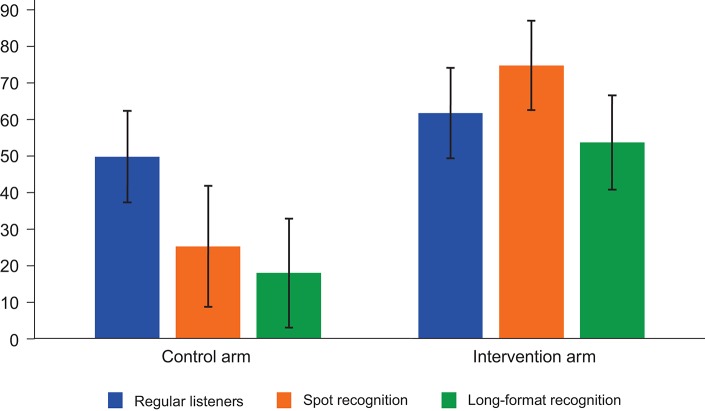
Radio Listenership and Campaign Recognition at Midline (%, 95% CI) Abbreviation: CI, confidence interval.

At baseline, according to interviews with household heads, around two-thirds of women in each arm had access to a radio in their household (Table 3). At midline, around half of women in each arm reported access to a radio in their household. Although reported household radio ownership was lower than at baseline, close to 80% of interviewed women in the intervention arm had access to a radio, either in the compound or in the household, and 62% were regular listeners, i.e., they reported listening to the radio in the past 7 days (Figure 3).

In the intervention arm, 75% of women reported recognizing at least 1 of the 2 spots played at the end of the interview, and 54% reported listening to the long-format program (Figure 3). Recognition of spots and of the long-format program was higher among regular radio listeners than among all women (88% for spots and 67% for the long-format program). In the control arm, 25% of women reported recognizing at least 1 of the 2 spots, and 18% reported listening to the long-format program (20% and 12%, respectively, when “contaminated” villages were excluded).

Three-quarters of women in the intervention arm reported recognizing at least 1 of 2 radio spots played for them.

### Change From Baseline in Self-Reported Behaviors

At midline, 43% of mothers overall reported that their child had suffered from one or more of the target childhood illnesses in the 2 weeks prior to interview. Period prevalence of these illnesses was similar in each arm: around 30% of children suffered from fever, 12% from diarrhea, and 8% from fast/difficult breathing. Most sick children for whom health care was sought went to a CSPS (92%). Only 10% went to a community health worker (CHW) and 2% or less to a hospital. Care seeking in private facilities was almost non-existent (6 cases only).

Table 4 presents the results of the intention-to-treat analysis, showing the prevalence of self-reported behaviors by arm at each survey and the corresponding “crude” and adjusted DiDs, i.e., the difference between arms in the change in prevalence from the baseline to the midline survey. Crude DiDs refer to the difference-in-difference without any adjustment for baseline prevalence or for confounder score.

**TABLE 4:** Changes From Baseline in Self-Reported Behaviors (Intention-to-Treat Analysis)

				Cluster-Level DiD Analysis				
					Adjusted for Baseline Prevalence		Adjusted for Baseline Prevalence and Confounder Score	
Behaviors	Survey	Control Arm No. (%)	Intervention Arm No. (%)	Crude DiD	DiD (95% CI)	*P* Value	DiD (95% CI)	*P* Value
**Maternal health**								
4 or more ANC visits	BS	2562 (50.8)	2470 (37.0)					
	MD	1012 (61.0)	1212 (48.4)	1.2	-3.5 (-17.8, 10.9)	.61	-5.6 (-23.7, 12.5)	.50
Saving money during the pregnancy	BS	2562 (62.8)	2474 (56.4)					
	MD	1012 (67.4)	1212 (69.8)	8.8	9.8 (1.0, 18.6)	.03	12.8 (1.4, 24.2)	.03
Health facility delivery	BS	2562 (81.8)	2470 (56.0)					
	MD	1012 (92.4)	1212 (65.3)	-1.3	1.1 (-11.0, 13.2)	.85	-1.0 (-12.6, 10.6)	.85
**Newborn health**								
Breastfeeding initiation within 1 hour after birth	BS	2556 (32.6)	2463 (26.8)					
	MD	1003 (31.2)	1194 (31.5)	6.1	8.5 (-6.7, 23.6)	.24	9.0 (-16.9, 34.9)	.46
First bath delayed for 24 hours or more after birth	BS	2556 (55.9)	2463 (49.3)					
	MD	1003 (66.0)	1194 (52.0)	-7.4	-5.5 (-16.3, 5.4)	.29	-3.4 (-19.1, 12.4)	.64
**Health care seeking in a health facility or with a CHW (2 weeks prior to interview)**								
Fever	BS	735 (63.7)	637 (50.2)					
	MD	777 (73.1)	744 (65.9)	6.3	4.2 (-7.9, 16.3)	.46	5.0 (-9.7, 19.6)	.47
Fast/difficult breathing	BS	302 (56.6)	381 (44.4)					
	MD	203 (70.9)	180 (62.8)	4.1	-2.9 (-27.0, 21.2)	.80	10.5 (-18.0, 39.1)	.43
Diarrhea	BS	559 (57.8)	514 (44.9)					
	MD	264 (65.5)	349 (65.0)	12.4	12.0 (-2.0, 26.1)	.09	17.5 (2.5, 32.5)	.03
**Treatment (2 weeks prior to interview)**								
ACT or quinine IM/IV for fever	BS	735 (17.3)	639 (16.9)					
	MD	797 (34.6)	766 (32.8)	-1.4	-4.1 (-12.7, 4.5)	.32	0.0 (-11.5, 11.5)	>.99
Antibiotic for fast/difficult breathing	BS	302 (28.2)	382 (27.0)					
	MD	210 (33.8)	188 (45.2)	12.6	13.8 (-7.9, 35.5)	.19	29.6 (3.5, 55.7)	.03
ORS or more liquids for diarrhea	BS	560 (41.1)	516 (30.6)					
	MD	274 (42.0)	354 (55.9)	24.4	14.9 (2.0, 27.8)	.03	9.5 (-5.6, 24.7)	.19
Homemade solutions for diarrhea	BS	560 (6.8)	516 (6.8)					
	MD	275 (6.2)	362 (8.3)	2.1	3.3 (-6.2, 12.9)	.46	3.2 (-10.5, 16.8)	.62
**Nutrition**								
Exclusive breastfeeding (day prior to interview, 0–5 months old)	BS	428 (44.6)	450 (42.4)					
	MD	323 (61.6)	361 (50.4)	-9.0	-10.3 (-24.3, 3.6)	.13	-8.7 (-28.2, 10.8)	.34
Complementary feeding (day prior to interview, 6–11 months old)	BS	418 (55.5)	411 (49.9)					
	MD	313 (57.8)	394 (52.3)	0.1	-3.3 (-17.4, 10.7)	.61	-10.0 (-27.6, 7.7)	.24
Growth monitoring (past 6 months, 0–23 months old)	BS	1525 (59.7)	1615 (51.1)					
	MD	1358 (66.3)	1513 (58.1)	0.4	0.1 (-11.1, 11.4)	.98	-2.6 (-17.3, 12.0)	.70
**Bed net use**								
Children under an ITN the night prior to interview	BS	2567 (60.3)	2475 (58.5)					
	MD	2585 (91.4)	2596 (87.8)	-1.8	-2.7 (-8.9, 3.5)	.35	-3.9 (-12.4, 4.7)	.34
Women under a bed net during their last pregnancy	BS	2560 (65.6)	2468 (62.5)					
	MD	2586 (79.2)	2594 (80.0)	3.9	2.1 (-5.3, 9.5)	.55	-0.6 (-10.6, 9.3)	.89
**Sanitation**								
Household latrine ownership	BS	2559 (19.5)	2458 (25.0)					
	MD	2585 (27.8)	2596 (35.2)	1.9	0.0 (-11.2, 11.3)	>.99	6.0 (9.3, 21.4)	.40
Safe disposal of children's last stools^a^	BS	2566 (14.1)	2475 (15.3)					
	MD	2562 (19.1)	2577 (21.2)	0.9	0.2 (-6.4, 6.8)	.95	2.4 (-7.0, 11.8)	.58
Hand washing with soap the last time women cleaned their child who defecated	BS	2535 (37.5)	2401 (36.4)					
	MD	2428 (45.6)	2426 (44.2)	-0.3	-1.6 (-21.0, 17.8)	.86	-10.5 (-35.9, 14.9)	.38

Abbreviations: ACT, artemisinin-based combination therapy; ANC, antenatal care; BS: Baseline survey; CHW, community health worker; CI, confidence interval; DiD, difference-in-difference; IM/IV, intramuscular/intravenous; MD: Midline survey; ORS, oral rehydration solution.

a Defined when the child used a latrine or when the stool was thrown into a latrine or buried.

Self-reported **care seeking for** **diarrhea** increased between baseline and midline by 17.5 percentage points more in the intervention arm than in the control arm, with some evidence for an effect of the campaign (adjusted DiD for baseline prevalence and confounder score, 17.5 percentage points; 95% CI, 2.5 to 32.5; *P* = .03) (Table 4). Self-reported **treatment with oral rehydration solution (ORS) or increased fluids** during an episode of diarrhea increased substantially in the intervention arm while it remained constant in the control arm (adjusted DiD for baseline prevalence, 14.9 percentage points; 95% CI, 2.0 to 27.8; *P* = .03), but the evidence for a difference between arms weakened after adjustment for confounder score (adjusted DiD for baseline prevalence and confounder score, 9.5 percentage points; 95% CI, -5.6 to 24.7; *P* = .19). No messages were broadcast on homemade solutions, and their use remained constant between surveys in each arm, with fewer than 10% of mothers mentioning use of homemade solutions.

The campaign had a positive effect on self-reported care seeking for diarrhea.

While the data on self-reported **care seeking for** **fast/difficult breathing** were inconclusive (adjusted DiD for baseline prevalence and confounder score, 10.5 percentage points; 95% CI, -18.0 to 39.1; *P* = .43), the proportion of children with fast/difficult breathing who were reported to have been **treated with an antibiotic** showed a much greater increase between surveys in the intervention arm compared with the control arm (adjusted DiD for baseline prevalence and confounder score, 29.6 percentage points; 95% CI, 3.5 to 55.7; *P* = .03).

The proportion of children with fast/difficult breathing reported to have received antibiotic treatment increased much more between surveys in the intervention arm than in the control arm.

There was no evidence for improved **care seeking for** **fever** (adjusted DiD for baseline prevalence and confounder score, 5.0 percentage points; 95% CI, -9.7 to 19.6; *P* = .47) or for **treatment of fever** associated with the campaign (adjusted DiD for baseline prevalence and confounder score, 0.0 percentage points; 95% CI, -11.5 to 11.5; *P* > .99).

While self-reported **saving during pregnancy** remained relatively constant between surveys in the control arm, it increased somewhat in the intervention arm (adjusted DiD for baseline prevalence and confounder score, 12.8 percentage points; (95% CI, 1.4 to 24.2; *P* = .03).

Whereas the broad pattern of results with respect to care seeking and treatment for the targeted childhood illnesses was positive, there was no evidence of an intervention effect on **feeding behaviors**: early initiation of breastfeeding (adjusted DiD for baseline prevalence and confounder score, 9.0 percentage points; 95% CI, -16.9 to 34.9; *P* = .46), exclusive breastfeeding (adjusted DiD for baseline prevalence and confounder score, -8.7 percentage points; 95% CI, -28.2 to 10.8; *P* = .34), and complementary feeding (adjusted DiD for baseline prevalence and confounder score, -10.0 percentage points; 95% CI, -27.6 to 7.7; *P* = .24).

For other target behaviors, including **bed net use** and **sanitation**, there was also no evidence that the campaign had an effect. Reported attendance to 4 or more ANC consultations, delivery in a health facility, bed net use, and latrine ownership increased in the intervention arm between surveys, but similar increases were observed in the control arm. Little or no change between surveys was observed in either arm in reporting of delayed bathing, growth monitoring, safe disposal of children’s stools, or hand washing with soap after cleaning a child’s bottom.

With respect to women living in “contaminated” villages (Gayeri control cluster) who were excluded from the per-protocol analysis, 80% belonged to the Gourmantche ethnic group, 68% were Catholic or Protestant, 46% had 2 or more children less than 5 years old, 66% had access to a radio in their household, and 55% lived less than 2 km away from a health facility. Other sociodemographic characteristics were typical of other women in the control arm. Excluding these women from the analysis, confounder score-adjusted DiDs for self-reported care seeking for diarrhea, fast/difficult breathing, and fever tended to be higher than the adjusted DiDs from the intention-to-treat analysis (adjusted DiD for baseline prevalence and confounder score, 22.0 percentage points; 95% CI, 6.93 to 37.0; *P* = .01); (17.3 percentage points; 95% CI, -10.3 to 44.9; *P* = .19); and (14.3 percentage points; 95% CI, -1.1 to 29.6; *P* = .07), respectively. For other behaviors, per-protocol analyses produced results similar to the intention-to-treat analyses (see supplementary material).

### Analysis Restricted To Regular Listeners and Dose-Response Analyses

Restricting the analysis to regular listeners produced similar results to results among all women mentioned above (data not shown). There was no evidence that the effect of the campaign varied with radio ownership (data not shown), but tests for effect modification had very low power due to small numbers of observations for some behaviors.

**FIGURE 4.**
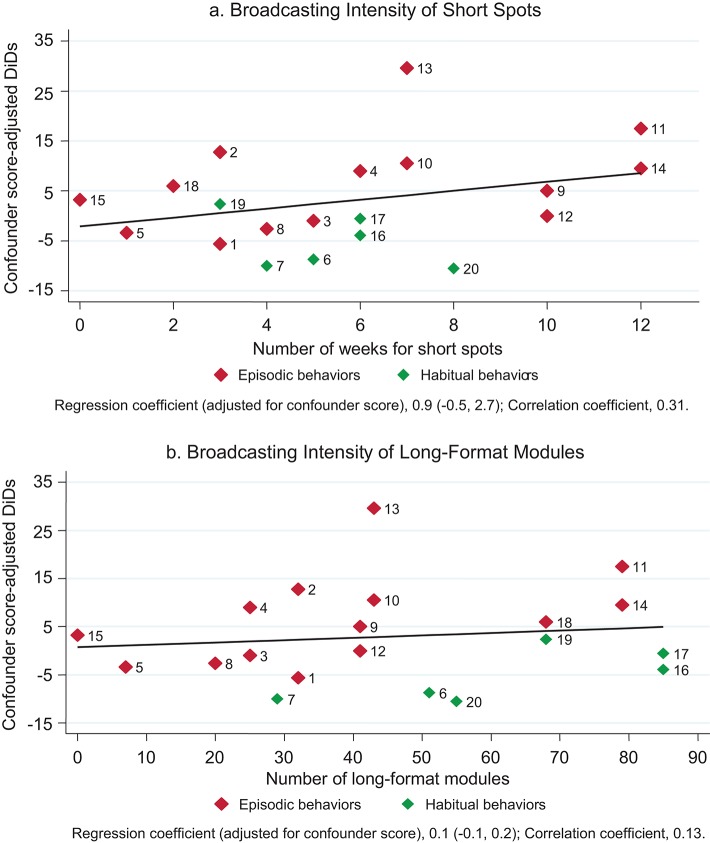
Correlation Between Changes in Targeted Behaviors and Broadcasting Intensity (Intention-to-Treat Analysis) Abbreviations: ACT/QN, artemisinin-based combination therapy/quinine; ANC, antenatal care; DiD, difference-in-difference; HMS, homemade solution; HWWS, hand washing with soap; ITN, insecticide-treated bed net; ORS, oral rehydration solution. Numbers in charts pertain to the targeted behaviors: (1) 4 or more ANC visits, (2) Savings-Pregnancy, (3) Facility delivery, (4) Breastfeeding within 1 h, (5) 1st bath 24 h or more, (6) Exclusive breastfeeding, (7) Complementary feeding, (8) Growth monitoring, (9) Care seeking: Fever, (10) Care seeking: Fast/difficult breathing, (11) Care seeking: Diarrhea, (12) ACT/QN-Fever, (13) Antibiotic-Fast/difficult breathing, (14) ORS/more liquids-Diarrhea, (15) HMS-Diarrhea, (16) ITN use-Children, (17) Bed net use-Pregnancy, (18) Latrine ownership, (19) Stools' safe disposal, (20) HWWS.

There was some suggestion of a positive correlation between the intensity of spots and reported behavior change prior to adjustment for confounder score (regression coefficient, 0.8 percentage point increase per week of spot; 95% CI, -0.1 to 1.7). Adjustment for confounder score made relatively little difference to the estimated regression coefficient (regression coefficient, 0.9 percentage point increase per week of spot) but resulted in a wider confidence interval (95% CI, -0.5 to 2.7) (Figure 4a). There was no evidence of correlation with the number of long-format modules broadcast (regression coefficient, 0.1 percentage point; 95% CI, -0.1 to 0.2) (Figure 4b).

### Change From Baseline in Routine Health Facility Data

Table 5 shows the absolute numbers of consultations for targeted health services in 40 and 37 primary health facilities located in the control and intervention arms, respectively. There was no statistical evidence for a difference between the 2 arms for any of the indicators (*P* ≥ .40), although the observed increase in all-cause under-5 consultations was much greater in the intervention arm (33% increase between 2011 and 2013) than in the control arm (17% increase).

Health facility data were consistent with a much greater increase in the number of all-cause under-5 consultations in the intervention arm than in the control arm.

**TABLE 5:** Number of Attendances for Targeted Health Services in Primary Health Facilities Located in Control (N = 40) and Intervention (N = 37) Arms, Based on Routine Health Facility Data, 2011 and 2013

	Arm	2011	2013	% Change From 2011 to 2013	*P* Value
4 ANC visits	Control	4,490	4,682	4	
	Intervention	6,283	6,723	7	.75
Health facility deliveries	Control	10,054	10,105	1	
	Intervention	11,911	12,928	9	.54
All-cause under-5 child consultations	Control	67,172	78,265	17	
	Intervention	78,892	104,535	33	.40

## DISCUSSION

After 20 months, the radio campaign in Burkina Faso appears to have reached a high proportion of the primary target population, with 75% of mothers in intervention areas reporting recognizing spots played at the end of the interview. However, a relatively high proportion of women reported recognizing spots in the control arm, too (25%). Although “contamination” is known to have occurred in Gayeri control cluster, the distances to the closest intervention radio station preclude population-level contamination in the other control clusters. “Courtesy” bias and confusion with other radio programs may explain the reported recognition in the control clusters. Some women in the intervention arm who reported recognizing spots may also have answered with “courtesy” or confused them with other messages.

Our findings are mixed with respect to the campaign’s effects on behavior. Among 19 target behaviors, there was some evidence of positive effects on self-reported appropriate family responses to diarrhea and fast/difficult breathing and saving money during pregnancy. Self-reported care seeking and home treatment for diarrhea increased more in the intervention arm, although there was no statistical evidence for the latter after controlling for confounder score. A relatively small number of mothers reported that their children had suffered from fast/difficult breathing and, consequently, results for behaviors related to this illness had wide confidence intervals. Nevertheless, the data are consistent with greater increases in self-reported care seeking and antibiotic treatment for this illness in the intervention arm. Routine health facility data are also consistent with these results, with a greater increase in all-cause under-5 child consultations in the intervention arm, but are inconclusive from a statistical perspective.

For other target behaviors, there was no evidence that the radio campaign had an effect. While some behaviors appear to have changed little between baseline and midline in either arm, others appear to have improved to similar degrees in each arm. There is some evidence from other sources^23^ of increases in ANC attendance, facility delivery, exclusive breastfeeding, and care seeking for fever over recent years, although these changes are not always as rapid as those we observed. The similar increases reported in each arm in antimalarial treatment might also be explained by a seasonal variation in health care providers’ treatment practices, the baseline having been performed in the dry season and the midline in November shortly after the last rains. In the case of bed net use, the results likely reflect effective national distribution in the summer of 2013 before the midline survey took place. Bed net ownership was almost universal at midline, with 99% of women reporting living in a household with at least 1 bed net. Latrine ownership increases may reflect in part the effects of latrine construction programs in various clusters.

The radio campaign had a positive effect on a few targeted behaviors, such as appropriate responses for diarrhea and fast/difficult breathing, but there was no evidence of an effect on other behaviors.

Why does the intervention appear to have had an impact on some behaviors but not others? First, intensity of the intervention is likely to be critical. Although the number of spots broadcast per day was high, on average 10 spots a day, and the long-format program was on air 5 days a week, the intensity allocated to each behavior varied substantially, from 1 week of spots for delayed bathing to 12 weeks of spots for management of diarrhea up to the month preceding the midline survey (Table 1). The dose-response analysis is consistent with those behaviors subject to the greatest number of weeks of spots tending to show the largest changes, although the statistical evidence for this is weak. There is no such pattern, however, for the number of long-format modules.

Another possible explanation for the mixed results may lie in the nature of the behaviors themselves. Changes may be difficult to achieve when they face habitual or normative practices that bear the weight of tradition and strong cultural beliefs.^24^ Such traditions and cultural beliefs are likely to vary from one setting to another. Perhaps more importantly, many preventive behaviors must be performed on a daily basis, with no immediately obvious benefit. Nutrition and hygiene-related behaviors, for example, share these characteristics and changing them may require more time and effort. This challenge to changing preventive behaviors may apply in many settings and across different behavior change approaches. In rural Burkina Faso, all behaviors for which we found some evidence for an intervention effect were episodic.

Behavior change may be difficult for habitual or normative practices such as those related to nutrition or hygiene.

Michie et al. (2011)^25^ have proposed a framework for characterizing behavior change interventions. This includes a behavioral model, in which “motivation,” “capability,” and “opportunity” interact to determine behavior. According to this framework and given the theory of change underpinning DMI’s campaign,^12^ one might speculate that the following mechanisms explain the observed changes in behavior. DMI’s messages, rather than providing information alone, use health-related storylines, which provide examples for people to aspire to, imitate, and elicit either positive or negative feelings about target behaviors. By combining information and entertainment, the campaign may act not only through the “capability” component of behaviors (knowledge) but also through “motivation,” by affecting both emotional responses and analytical decision making. In addition, the immediate social circle of women and other members of their community were also exposed to the campaign. While husbands influence birth preparedness through permitting (or not) expenditures,^26^ female family members, such as mothers-in law, aunts, or grandmothers, are frequently present at the time of birth, provide guidance during the first months of the baby’s life, and influence breastfeeding practices.^27^^,^^28^ Beside beliefs about disease etiology and perceived severity of illnesses, family members also influence decisions about whether and where to seek care in the event of childhood illnesses.^29^ By reaching a large audience, the campaign may also have triggered dialogue in the community and brought changes in the social norms or “social opportunity” component of behaviors, defined as the “cultural milieu that dictates the way people think about things.”^25^

On the other hand, the “physical opportunity” component of behaviors, defined as the external conditions that make behavior change possible,^25^ was unaffected by the campaign and this needs to be considered when interpreting results. In 2010, Burkina Faso ranked 161 of 169 countries in UNDP’s Human Development Index with 44% of the population living below the poverty line and 77% living in rural areas.^13^ The poverty of the studied population is therefore likely to be an important barrier to changes in some behaviors, such as nutrition or sanitation-related behaviors. In addition, rural populations, largely dependent on subsistence agriculture, are vulnerable to food insecurity, the last crisis having occurred in 2012. In 2013, Burkina Faso ranked 65 of 78 on the Global Hunger Index.^30^ In this context, improving complementary feeding practices may require more practical support. Access to treatment is another potential limitation. For example, at midline, only 43% and 31% of surveyed villages in the control and intervention arms, respectively, had ORS available within the village itself (either at a primary health facility or through a CHW).

Finally, it should also be borne in mind that in this campaign exposure is largely passive, although the long-format programs did give listeners the opportunity to phone in. Other behavior change interventions have often used interpersonal communication that involves face-to-face interaction between health promoters and caregivers. Face-to-face encounters provide some opportunity to tailor information to caregivers’ needs and to use persuasion and social influence.^8^ It has been suggested that programs in which mass media is part of a multifaceted intervention strategy are more likely to be successful than mass media alone.^9^^,^^10^ However, such programs are generally far more costly to implement effectively on a large scale.

Of the 32 evaluations of mass media campaigns identified by Naugle et al. (2014)^10^ that relied on “moderate” to “stronger” designs, all but 6 were reported to show some evidence of positive effects on child survival-related behaviors. However, only 2 evaluations reported using randomized designs, one of which randomized only 4 clusters, and the authors also note the potential for publication bias. All but 6 evaluated programs that included interpersonal communication components, e.g., training of health workers or volunteers, or implementing community-based activities, but none was able to disentangle the impact of different components. The results of the 6 evaluations of programs that used mass media alone were generally consistent with positive effects but had important design limitations.

In Peru and the Philippines, vaccination coverage rates were reported to have improved by 10 to 20 percentage points following radio and TV campaigns, but no concurrent control data were available.^31^ In Central Java, Indonesia, a radio campaign was accompanied by improvements in reported fluid intake during diarrhea, but a similar change was observed in control areas; Hornik (2002)^31^ concluded that the change was probably unrelated to the campaign. In Bolivia, a new brand of nutritional supplement for women was promoted through a radio and TV campaign, with 11% of women at endline reporting having taken the supplement at least once.^32^ A radio and TV campaign, accompanied by SMS reminders, in Cameroon was associated with a 12 percentage point increase in bed net use among children under 5 years compared with the matched control group.^33^ Finally, Jaramillo (2001)^34^ reported a transient increase in the number of individuals being tested for tuberculosis in Cali, Colombia, coinciding with a TV and radio campaign. No such increase was seen in a control area, which did not receive the campaign.

Thus, the evidence base for the effectiveness of mass media in improving child survival, whether alone or with interpersonal components, is very limited, and it is impossible to make strong assertions about the relative impact of different strategies. To our knowledge, our study is the first cluster randomized trial to investigate and present evidence that a mass community radio campaign alone can change some health-related behaviors in a low-income setting.

### Limitations

Several limitations of this study must be recognized. First, although clusters were randomly allocated to receive the intervention, there were some important differences at baseline between intervention and control arms. Baseline imbalance is not uncommon when only a few clusters are randomized, and we sought to control for these differences by creating a confounder score. However, we cannot exclude the possibility that this imbalance resulted in some bias in our comparisons of intervention and control clusters. Second, the evaluation largely relied on self-reported behaviors whose accuracy may be questioned. Some behaviors such as place of delivery, recognition of fever, and treatment with ACTs may be more accurately reported than behaviors occurring immediately after birth or recognition and antibiotic treatment of pneumonia.^35^ The length of the questionnaire, 40 minutes on average, may also have resulted in interview fatigue affecting women’s recall at the end of the interview. In addition, socially desirable behaviors may be overreported.^36^^,^^37^ When possible, we sought documentary evidence to reduce the probability of misreporting. For example, fieldworkers asked women whether they had a prescription or a package for any treatments given to their child. At both surveys, supporting evidence was available for about 70% of ACT treatments given to febrile children and oral antibiotics given to children with fast/difficult breathing. In addition, routine health facility data from the Ministry of Health are consistent with the observed changes from baseline to midline in self-reported service-dependent behaviors. Nevertheless, we cannot exclude the possibility that DMI’s campaign itself could have increased overreporting of target behaviors in the intervention clusters, although if reporting bias did occur to an important degree one might have expected to see positive results across a wider range of behaviors. Third, the power and precision of the trial is limited by the relatively small number of clusters that could be randomized, and this limits our ability to detect modest changes in behaviors. Fourth, the baseline and midline surveys were not performed at exactly the same period of the year, with the baseline performed between December and March and the midline survey performed in November. Seasonal variation in behaviors may explain some of the changes observed between baseline and midline but should not have confounded the comparison between intervention and control clusters. Fifth, although major co-interventions (Table 2) were implemented in similar numbers of clusters per arm, we did not collect data on their intensity and quality. Sixth, we excluded towns and large villages where access to television may limit the effect of a campaign delivered using local community radio stations. This exclusion limits, to some extent, the generalizability of our findings although it does not affect their internal validity (though it is unlikely that the addition of television messages would reduce the impact of the campaign). Lastly, we have examined multiple behaviors (19), but the differences between intervention and control arms were below the conventional cut-off point of *P* = .05 after adjustment for only some (3) of the behaviors. All these limitations mandate a cautious interpretation of our results.

## CONCLUSION

DMI’s *Saturation+* approach to designing and implementing a mass radio campaign had positive effects at midline on some maternal and child health behaviors such as saving money during pregnancy and appropriate family responses to diarrhea and fast/difficult breathing. However, there was no statistical evidence that the campaign had an effect on ANC consultations, facility delivery, delayed bathing, early initiation of breastfeeding, care seeking for and treatment of fever, bed net use, nutrition, or sanitation-related behaviors. Dose-response analysis of broadcasting intensity showed that behaviors associated with the greatest number of weeks of broadcasted spots tended to have the largest changes, although there is weak evidence of such an effect from a statistical perspective. The impact of the radio campaign on child mortality will be evaluated at endline.

## Supplementary Material

Supplementary Material

## Notes

**Peer Reviewed**
