# Supplementary material for: Behavior Change After 20 Months of a Radio Campaign Addressing Key Lifesaving Family Behaviors for Child Survival: Midline Results From a Cluster Randomized Trial in Rural Burkina Faso
Source: Glob Health Sci Pract. 2015 Nov 3;3(4):557–76. doi: 10.9745/GHSP-D-15-00153 (PMC4682582; doi:10.9745/GHSP-D-15-00153)
Supplement: Supplementary Material [file supp_3_4_557__index.html]

Supplementary Material 

# Behavior Change After 20 Months of a Radio Campaign Addressing Key Lifesaving Family Behaviors for Child Survival: Midline Results From a Cluster Randomized Trial in Rural Burkina Faso

## GHSP-D-15-00153 Supplementary Material

Sarrassat et al. doi: 10.9745/GHSP-D-15-00153

- Supplementary Material - Sarrassat et al. doi: 10.9745/GHSP-D-15-00153
