# Supplementary material for: Behavior Change After 20 Months of a Radio Campaign Addressing Key Lifesaving Family Behaviors for Child Survival: Midline Results From a Cluster Randomized Trial in Rural Burkina Faso
Source: Glob Health Sci Pract. 2015 Nov 3;3(4):557–76. doi: 10.9745/GHSP-D-15-00153 (PMC4682582; doi:10.9745/GHSP-D-15-00153)
Supplement: Supplementary Material [file GHSP-D-15-00153_15-00153-Sarrassat-Supplementary-Table.pdf]

Sarrassat S, Meda N, Ouedraogo M, Some H, Bambara R, Head R, et al. Behavior change after 20 months of a mass media campaign addressing key lifesaving family behaviors for child survival: midline results from a cluster randomized trial in rural Burkina Faso. Glob Health Sci Pract. 2015. Available from: http://dx.doi.org/10.9745/GHSP-D-15-00153

SUPPLEMENTARY TABLE. Changes From Baseline in Self-Reported Behaviors (Per Protocol Analysis)

		Control Arm		Intervention Arm			Cluster-Level DiD Analysis							
							Adjusted for Baseline Prevalence				Adjusted for Baseline Prevalence and Confounder Score			
	Survey	Total	%	Total	%	"Crude" DiD	DiD	95% CI		*P*	DiD	95% CI		*P*
**Maternal health**														
4 or more ANC visits	BS	2562	**50.8**	2470	**37.0**									
	MD PP	912	**60.1**	1212	**48.4**	2.1	**-4.1**	-19.0	10.8	0.557	**-7.3**	-25.9	11.3	0.401
Saving money during the pregnancy	BS	2562	**62.8**	2474	**56.4**									
	MD PP	912	**68.4**	1212	**69.8**	7.8	**8.1**	-0.4	16.7	0.061	**7.6**	-3.9	19.1	0.173
Health facility delivery	BS	2562	**81.8**	2470	**56.0**									
	MD PP	912	**93.3**	1212	**65.3**	-2.2	**3.1**	-8.0	14.2	0.555	**1.4**	-9.6	12.5	0.781
**Newborn health**														
Breastfeeding initiation within 1 hour after birth	BS	2556	**32.6**	2463	**26.8**									
	MD PP	906	**26.5**	1194	**31.5**	10.8	**8.2**	-8.2	24.7	0.295	**7.7**	-20.4	35.8	0.553
First bath delayed for 24 hours or more after birth	BS	2556	**55.9**	2463	**49.3**									
	MD PP	906	**70.0**	1194	**52.0**	-11.4	**-5.6**	-16.3	5.2	0.280	**-3.6**	-19.2	12.1	0.624
**Health care seeking in a health facility or with a CHW (2 weeks prior to interview)**														
Fever	BS	735	**63.7**	637	**50.2**									
	MD PP	704	**72.4**	744	**65.9**	7.0	**8.6**	-5.7	23.0	0.213	**14.3**	-1.1	29.6	0.065
Fast/difficult breathing	BS	302	**56.6**	381	**44.4**									
	MD PP	161	**72.1**	180	**62.8**	2.9	**-1.0**	-26.7	24.8	0.936	**17.3**	-10.3	44.9	0.193
Diarrhea	BS	559	**57.8**	514	**44.9**									
	MD PP	229	**66.4**	349	**65.0**	11.5	**14.1**	-1.5	29.7	0.072	**22.0**	6.9	37.0	0.009
**Treatment (2 weeks prior to interview)**														
ACT or quinine IM/IV for fever	BS	735	**17.3**	639	**16.9**									
	MD PP	724	**34.0**	766	**32.8**	-0.8	**-1.6**	-12.2	9.0	0.745	**7.1**	-5.0	19.1	0.220
Antibiotic for fast/difficult breathing	BS	302	**28.2**	382	**27.0**									
	MD PP	168	**38.7**	188	**45.2**	7.7	**14.7**	-7.6	37.0	0.175	**31.9**	5.8	58.1	0.021
ORS or more liquids for diarrhea	BS	560	**41.1**	516	**30.6**									
	MD PP	239	**42.3**	354	**55.9**	24.1	**14.4**	1.5	27.4	0.032	**8.5**	-6.5	23.5	0.234
Homemade solutions for diarrhea	BS	560	**6.8**	516	**6.8**									
	MD PP	240	**5.0**	362	**8.3**	3.3	**4.7**	-4.6	14.1	0.289	**7.1**	-6.0	20.1	0.255
**Nutrition**														
Exclusive breastfeeding (day prior to interview, 0–5 months old)	BS	428	**44.6**	450	**42.4**									
	MD PP	291	**59.1**	361	**50.4**	-6.5	**-7.4**	-21.5	6.6	0.268	**0.3**	-17.6	18.2	0.969
Complementary feeding (day prior to interview, 6–11 months old)	BS	418	**55.5**	411	**49.9**									
	MD PP	290	**56.9**	394	**52.3**	1.0	**-3.6**	-17.9	10.7	0.592	**-10.6**	-28.5	7.2	0.215
Growth monitoring (past 6 months, 0–23 months old)	BS	1525	**59.7**	1615	**51.1**									
	MD PP	1210	**64.5**	1513	**58.1**	2.2	**-0.6**	-12.8	11.6	0.916	**-5.0**	-20.4	10.4	0.486

Sarrassat S, Meda N, Ouedraogo M, Some H, Bambara R, Head R, et al. Behavior change after 20 months of a mass media campaign addressing key lifesaving family behaviors for child survival: midline results from a cluster randomized trial in rural Burkina Faso. Glob Health Sci Pract. 2015. Available from: http://dx.doi.org/10.9745/GHSP-D-15-00153

		Control Arm		Intervention Arm			Cluster-Level DiD Analysis							
							Adjusted for Baseline Prevalence				Adjusted for Baseline Prevalence and Confounder Score			
	**Survey**	**Total**	**%**	**Total**	**%**	**"Crude" DiD**	**DiD**	**95% CI**		***P***	**DiD**	**95% CI**		***P***
**Bed net use**														
Children under an ITN the night prior to interview	BS	2567	**60.3**	2475	**58.5**									
	MD PP	2333	**91.1**	2596	**87.8**	-1.5	**-3.0**	-9.4	3.4	0.328	**-4.6**	-13.3	4.2	0.274
Women under a net during their last pregnancy	BS	2560	**65.6**	2468	**62.5**									
	MD PP	2334	**78.2**	2594	**80.0**	4.9	**1.9**	-5.7	9.4	0.597	**-1.3**	-11.3	8.8	0.786
**Sanitation**														
Household latrine ownership	BS	2559	**19.5**	2458	**25.0**									
	MD PP	2333	**30.6**	2596	**35.2**	-0.9	**-0.7**	-12.0	10.5	0.889	**3.8**	-12.1	19.7	0.605
Safe disposal of children's last stools[a]	BS	2566	**14.1**	2475	**15.3**									
	MD PP	2310	**20.0**	2577	**21.2**	0.0	**0.1**	-6.4	6.7	0.971	**2.2**	-7.3	11.6	0.619
Hand washing with soap the last time women cleaned their child who defecated	BS	2535	**37.5**	2401	**36.4**									
	MD PP	2207	**44.3**	2426	**44.2**	1.0	**-2.6**	-22.8	17.6	0.785	**-13.4**	-39.1	12.3	0.273

Abbreviations: ACT, artemisinin-based combination therapy; ANC, antenatal care; BS: Baseline survey; CHW, community health worker; CI, confidence interval; DiD, difference-in-difference; IM/IV, intramuscular/intravenous; MD: Midline survey; ORS, oral rehydration solution.

[a] Defined when the child used a latrine or when the stool was thrown into a latrine or buried.
